# Using Auditory Characteristics to Select Hearing Aid Compression Speeds for Presbycusic Patients

**DOI:** 10.3389/fnagi.2022.869338

**Published:** 2022-06-30

**Authors:** Yi Zhang, Jing Chen, Yanmei Zhang, Baoxuan Sun, Yuhe Liu

**Affiliations:** ^1^Plastic Surgery Hospital, Chinese Academy of Medical Sciences and Peking Union Medical College, Beijing, China; ^2^School of Electronics Engineering and Computer Science, Peking University, Beijing, China; ^3^Department of Otorhinolaryngology Head and Neck Surgery, Peking University First Hospital, Beijing, China; ^4^Widex Hearing Aid (Shanghai) Co., Ltd., Shanghai, China; ^5^Department of Otolaryngology Head and Neck Surgery, Beijing Friendship Hospital, Capital Medical University, Beijing, China

**Keywords:** presbycusis, hearing aid, temporal modulation, speech recognition in noise, decision tree

## Abstract

**Objectives:**

This study aimed to select the optimal hearing aid compression speeds (fast-acting and slow-acting) for presbycusic patients by using auditory characteristics including temporal modulation and speech-in-noise performance.

**Methods:**

In total, 24 patients with unilateral or bilateral moderate sensorineural hearing loss who scored higher than 21 on the Montreal Cognitive Assessment (MoCA) test participated in this study. The electrocochleogram (ECochG) results, including summating potentials (SP) and action potentials (AP), were recorded. Subjects' temporal modulation thresholds and speech recognition at 4 individualized signal-to-noise ratios were measured under three conditions, namely, unaided, aided with fast-acting compression (FAC), and aided with slow-acting compression (SAC).

**Results:**

The results of this study showed that modulation discrimination thresholds in the unaided (−8.14 dB) and aided SAC (−8.19 dB) conditions were better than the modulation thresholds in the FAC (−4.67 dB) conditions. The speech recognition threshold (SRT75%) for FAC (5.21 dB) did not differ significantly from SAC (3.39 dB) (*p* = 0.12). A decision tree analysis showed that the inclusion of the AP, unaided modulation thresholds, and unaided SRT75% may correctly identify the optimal compression speeds (FAC vs. SAC) for individual presbycusic patients with up to 90% accuracy.

**Conclusion:**

Both modes of compression speeds improved a presbycusic patient's speech recognition ability in noise. The SAC hearing aids may better preserve the modulation thresholds than the FAC hearing aids. The measurement of AP, along with the unaided modulation thresholds and unaided SRT75%, may help guide the selection of optimal compression speeds for individual presbycusic patients.

## Introduction

Hearing affects people's quality of life. Presbycusis, or age-related hearing loss (ARHL), is a degenerative change of the auditory system associated with aging, often accompanied by decreased speech recognition of noise (Gates and Mills, 2005; Liu and Yan, 2007). Presbycusis not only affects daily communication and independent living but also increases the risk of cognitive decline resulting in dementia (Fortunato et al., 2016; Su et al., 2017; Bowl and Dawson, 2019). At present, hearing aids and cochlear implants are the only intervention measures to improve the hearing ability of elderly hearing-impaired patients (Sprinzl and Riechelmann, 2010). However, due to individual differences, hearing aids may not always provide the most optimal speech intelligibility for all individual patients. A strategy that considers individual characteristics in the selection of optimal hearing aid may be beneficial.

A major complaint from presbycusic patients is poor speech understanding in noise (speech in noise or SIN). Previous studies proposed that the reduced temporal processing ability of the elderly hearing-impaired listeners may account for some of the difficulties (Abel and Hay, 1996; Phillips, 1999; Wingfield et al., 2006; Anderson et al., 2012; Sergeyenko et al., 2013; Bramhall et al., 2015; Rance and Starr, 2015; Han and Dimitrijevic, 2020; Lad et al., 2020; Luo and Ding, 2020; Shader et al., 2020). Temporal resolution refers to the ability of the auditory system to respond to rapid changes in the acoustic signal. Temporal information can be divided into a temporal fine structure (TFS), periodicity, and temporal envelope (ENV). Several studies showed that the reserve of ENV can keep the sound naturalness, and TFS may be related to melody, tonal perception, and speech recognition in noise (Moon and Hong, 2014).

The inner hair cells of the cochlea are connected with the auditory nerve fibers through ribbon synapses. In mammals, auditory nerve fibers can be broadly divided into two types based on spontaneous discharge rate (SR), namely, low SR fibers and high SR fibers, which accounted for 40 and 60% of the total nerve fibers, respectively (Mohrle et al., 2016). High SR fibers have a lower threshold and play a leading role when sound intensity approaches the behavioral auditory threshold, and their discharge rate saturates at 20–30 dB above the threshold. However, low SR fibers have a higher threshold and a wider dynamic range and are helpful for sound recognition in noise (Profant et al., 2019). Animal studies have shown that noise exposure and aging lead to the loss of acoustic nerve fibers, especially low SR fibers, without significant threshold shifts (Frisina and Frisina, 1997). Animal experiments and computer simulation results suggest that the loss of low SR fibers affects the time coding of the sound envelope at suprathreshold levels (Grose and Hall, 2006). A study by Otte et al. (1978) showed that humans lose about 2,100 auditory neurons every 10 years. Studies found that the retainment of only 10–20% of the inner hair cells can keep a normal audiometric threshold, but a smaller percentage of fiber loss can lead to the decline of speech recognition ability (Lobarinas et al., 2013). Thus, listeners with degraded temporal resolution will have difficulty in speech recognition in challenging environments, even if they do not have hearing loss or difficulty in quiet environments (Grose and Mamo, 2010; Jayakody et al., 2018). Temporal resolution decreases with age (Queiroz et al., 2010; Lister et al., 2011; Fostick and Babkoff, 2013; Ozmeral et al., 2016). Thus, the elderly listeners will have difficulty with tasks such as temporal modulation discrimination, which measures a listener's ability to distinguish how much fluctuation in the intensity of a signal (or modulation depth) can be discriminated (Herrmann et al., 2019; Luo et al., 2020; Zhou et al., 2020; Mepani et al., 2021).

Bharadwaj et al. reported that in animal experiments, the loss of lower SR fibers led to a reduction in the supra-threshold amplitude of the ABR I wave (Bharadwaj et al., 2014; Mohrle et al., 2016). Other studies also showed that the amplitude of ABR wave I and action potential (AP) were sensitive measurements in potential noise-induced cochlear synapse disease (Liberman et al., 2016; Mehraei et al., 2016; Lobarinas et al., 2017; Valderrama et al., 2018). AP represents the total activity of auditory nerve fibers connected with hair cells, and the first negative wave of AP is defined as N1, which is the same component as ABR wave I and originates from the distal portion of the auditory nerve (Moller and Jannetta, 1983). Studies suggested that cochlear synaptopathy influences the connection between inner hair cells (IHC) and auditory nerves, resulting in low SR fiber dysfunction and thus reducing speech perception in noise (Furman et al., 2013). Chen et al. (2021) studied the effect of cochlear synaptopathy on presbycusis using ECochG; they found that some presbycusic patients may have cochlear synaptopathy, manifested by lower AP amplitude, causing speech perception in noise dysfunction. Since in sensorineural hearing loss, the ABR wave I often disappear, AP may be a viable measure to reflect the condition of the low SR fibers and cochlear synapses.

Souza reported that the temporal resolution of the individual listeners could affect their speech recognition (Souza, 2000). As hearing aids could alter the temporal and spectral characteristics of the input signals reaching the listener's auditory system, it is reasonable to expect that the type of compression processing in a hearing aid could affect the aided temporal resolution and/or aided speech in noise ability. Today's hearing aid compression patterns can be broadly classified into fast-acting compression (FAC) and slow-acting compression (SAC) types. Fast-acting compression hearing aids typically have attack times under 10 ms and release times between 5 and 200 ms (Kuk and Hau, 2017). They are also called syllabic compressors because the gain change in such devices follows the rapid intensity level changes between syllables of speech. A rationale for FAC is to ensure audibility and that the output sound intensity is within the residual hearing range of the hearing-impaired listeners. In so doing, FAC reduces the intensity contrasts between the louder and softer parts of the input signals (i.e., reduces modulation or increases smearing). SAC hearing aids typically use longer attack times (5–100 ms) and release times (as long as 2 s, but in some hearing aids, it can be as long as 20 s). This longer time constant maintains the intensity contrasts of the input signal (i.e., less smearing). On the contrary, the longer release time in the SAC may not provide sufficient gain to softer sounds following a more intense sound and loss of audibility may ensue.

There have been numerous experimental studies on the effect of FAC and SAC hearing aids. The majority of the studies reported that the sound quality of SAC hearing aids is generally preferred over FAC hearing aids (Korhonen et al., 2021). The results are mixed when speech recognition was considered. Some researchers believed that FAC can retain TFS better than SAC during the dip period of background noise. This helps the listeners to extract the target speech information (Festen and Plomp, 1990; Vestergaard et al., 2011; Kwon et al., 2012; Ozmeral et al., 2012). For example, Gatehouse et al.'s study on ten normal-hearing native-English speaking listeners found that the optimal compression speed varies from individual to individual, but most people get better speech recognition in noise with FAC than SAC (Gatehouse et al., 2006a,b). Moore et al. studied the relative benefits of SAC and FAC in 2-talker babble noise. When the direction of the speech signal and noise signal was different, the speech recognition ability of SAC was slightly better than that of FAC (Moore et al., 2010). Reinhart and Souza also found that for a high compression ratio, SAC resulted in better speech recognition (Reinhart and Souza, 2016). These conflicting results may be due to differences in materials, parameters of compression, and outcome measures used in different studies, and partly caused by individual differences among listeners. Souza reported that temporal resolution will influence speech recognition (Souza, 2000), and SAC can preserve the temporal waveform better than FAC (Kuk and Hau, 2017). So, we inferred that unaided temporal resolution may influence the outcome of the comparison between compression speeds. If the listeners have a good unaided temporal resolution, to begin with, they may not be as negatively affected by FAC (from the smearing); thus, no difference between FAC and SAC may result. If listeners have a poor unaided temporal resolution, they may be more negatively affected by FAC and less by SAC. Therefore, we suggest that measuring the unaided temporal resolution and the unaided speech in noise ability of the individual may offer insight into whether the patient may benefit more from FAC or SAC.

In this study, we plan to evaluate the effect of FAC and SAC on presbycusic patients' temporal modulation discrimination thresholds and speech in noise ability. These threshold measures will be combined with other individual audiometric characteristics in a decision tree analysis in order to help to preselect the optimal compression speed for better-aided speech recognition in noise.

## Methods

### Participants

The current dataset was based on 24 patients (9 male patients and 15 female patients, average age = 77 years) who were treated at Peking University First Hospital for hearing problems between March 2019 and June 2021. All patients have signed informed consent. Mandarin was their native language. Patients completed their medical case history intake and standard audiometry [including pure tone audiometry (PTA) and speech audiometry; electrocochleogram was also measured]. The Montreal Cognitive Assessment (MoCA) was also administered to rule out significant cognitive impairment.

**Figure 1:**
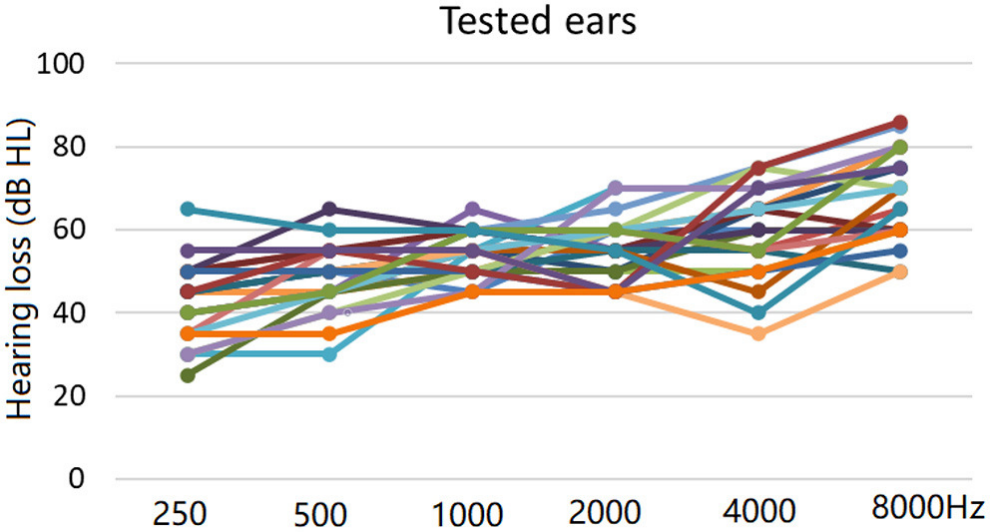
Individual audiometric thresholds in test ears (13 left ears and 11 right ears) using pure tone audiometry.

The demographic details and audiometry results are shown in Table 1. Audiometric thresholds of subjects' tested ears are shown in Figure 1.

**Table 1:** Demographic details and audiometry results of 24 subjects.

Age (year), median (Q1, Q3)	75.7, 77 (73.5, 81)
Male, *n* (%)	9 (37.5)
PTA (dB HL), median (Q1, Q3)	52.6, 51.7 (50.8, 55)
MoCA (point), median (Q1, Q3)	24.5, 25.5 (22.5, 27)
ECochG (-SP/AP %), median (Q1, Q3)	39.8, 38.5 (24.5, 57)
AP, median (Q1, Q3)	0.756, 0.745 (0.415, 1.023)

### Selection and Fitting of Test Hearing Aids

Two hearing aids with different modes of compression speeds were compared. The first was a 15-channel hearing aid that primarily uses SAC. It uses an adaptive attack time of <2 s and a release time of <20 s. The second was a 16-channel hearing aid that was used in the “syllabic compression” mode during this study (typically an attack time of <5 ms and a release time of <50 ms). Other than the compression algorithm, all other features within the hearing aids were deactivated during the study. Both hearing aids were fitted to the NAL-NL2 target by the same experimenter and verified using real-ear measurement to ensure that the target output between the two hearing aids was matched to within 3 dB at 500–4,000 Hz.

Because subjects were patients seeking hearing treatment, and binaural hearing aids, despite the clinicians' recommendations, were not a common practice in China, subjects were fitted and studied monaurally in the ear with a moderate-to-severe hearing loss. If both ears had moderate-to-severe sensorineural hearing loss, the test ear was randomly selected. The order of hearing aids tested was randomized. Subjects were blinded to the test hearing aids.

### Test and Stimulus Conditions

Temporal resolution was measured using a temporal modulation discrimination test written by the School of Electronics Engineering and Computer Science at Peking University. The test stimuli were cosine modulated sinusoids with a carrier frequency at 1 kHz and a duration of 600 ms. The default modulation rate for the amplitude modulation was 4 Hz. The standard stimuli were modulated at a modulation depth of −15 dB. The target modulation depth was initially set to −3 dB and adaptively varied in 2 dB and then 1 dB steps using a two-down one-up rule. The RMS level of the standard and target stimuli was normalized and roved at ±2 dB. A three alternative forced choice (3AFC) paradigm was used in which subjects selected the interval that sounded different. A modulation threshold of “0” would suggest 100% modulation or poor temporal resolution, whereas a more negative modulation threshold (e.g., −30 dB) would suggest a better temporal resolution. The unaided modulation threshold was measured at 85 dB SPL, while the aided thresholds were measured at a stimulus level of 80 dB SPL.

Speech recognition in noise was evaluated using the computer-aided Chinese speech audiometry platform (Ji et al., 2011a,b; Xi et al., 2012). Subjects identified the target sentence in 4-talker babble noise. Four customized signal-to-noise ratio (SNR) conditions (ranging from −12 dB to 18 dB) were used to obtain as broad a representation of the individual performance-intensity (P-I) function as possible in each test condition. That is, we attempted to test SNRs that yielded ≥50% speech recognition and that yielded <50% speech recognition. The target speech (sentence) and background (4-talker) were presented directly in front of the subject at a speech level of 85 dB SPL in the unaided mode and 80 dB SPL in the aided mode.

### Procedures

The medical case history intake and standard audiometry (including PTA and speech audiometry) were first performed along with ECochG. MoCA was administered to ensure no significant cognitive impairment. The two test hearing aids were then fitted before their amplitude modulation discrimination thresholds and speech-in-noise performance was measured under three conditions, namely, unaided, aided with FAC, and aided with SAC, in random order. Patients are blinded to the identity of the hearing aids. After the initial decision tree analysis, another 10 listeners (see later) were enrolled to verify the accuracy of the decision tree prediction.

Testing on speech recognition, amplitude modulation was conducted in a soundproof room with the stimuli presented directly in front of the subject by a soundbox. Stimuli intensity was measured by a sound pressure meter at 85 dB SPL during the unaided condition and 80 dB SPL in the aided condition with FAC and SAC (OTO suite was used as the test computer system). Subjects were tested monaurally with the non-test ear occluded with an earplug (made by OHRFRIEDEN with attenuation factor < 32 dB). Amplitude modulation discrimination thresholds were determined first, followed by a 10-min break before the speech in noise measurement was conducted. During each test, subjects were tested in the unaided, aided with FAC, and aided with SAC conditions in random order.

### Statistical Analysis

All the data were normally distributed, and standard independent *t*-tests were conducted. Python3.8 is used for statistical analysis based on sklearn and stats models. *P* < 0.05 is considered to be statistically significant.

### Ethics Statement

All protocols for this study were conformed to the Declaration of Helsinki and approved by the Biomedical Research Committee of the Peking University First Hospital (2020-219). The patients/participants provided their written informed consent to participate in this study.

## Results

### Temporal Amplitude Modulation Thresholds

**Figure 2:**
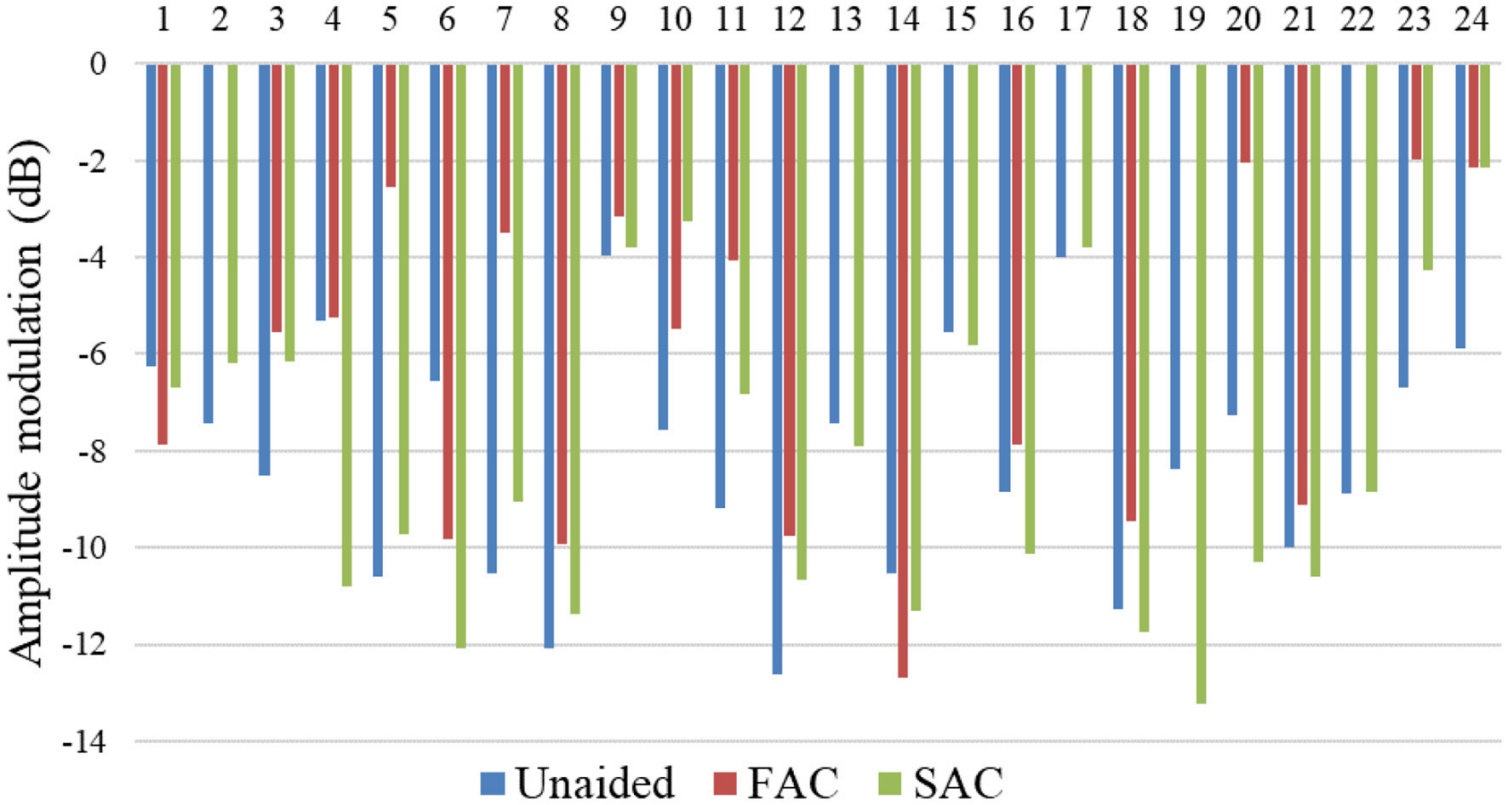
Temporal modulation discrimination thresholds measured across participants.

The individual temporal modulation thresholds are summarized in Figure 2. The mean unaided modulation threshold was −8.14 dB. The mean aided modulation threshold using FAC was −4.67 dB and that for SAC was −8.19 dB. As a reminder, a smaller modulation threshold reflects better temporal resolution.

**Figure 3:**
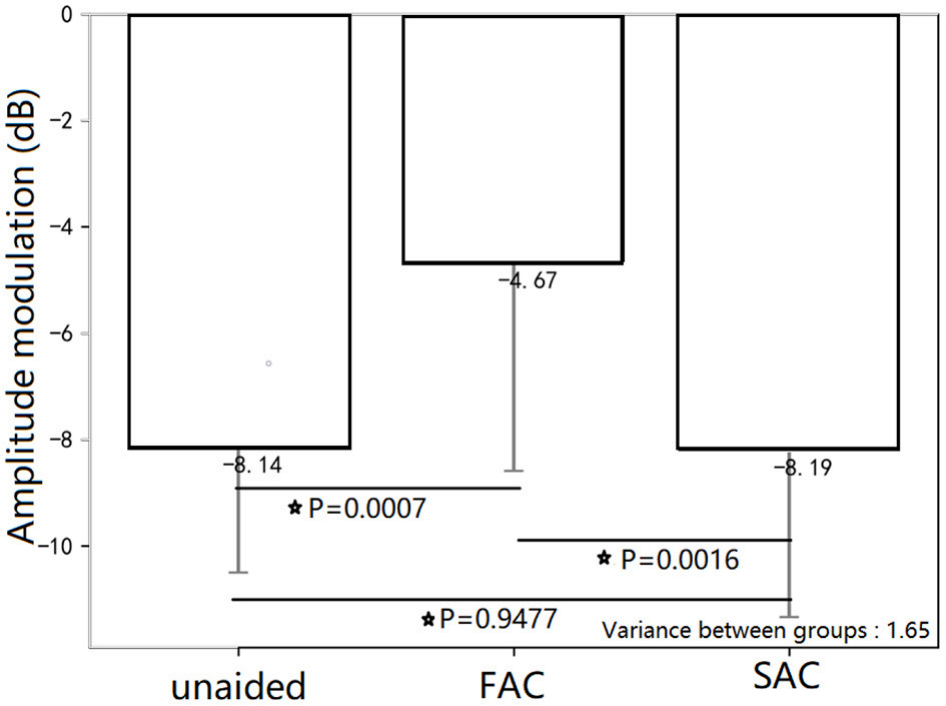
Mean temporal modulation discrimination thresholds for the three HA conditions. The unaided temporal modulation thresholds and the aided temporal modulation thresholds in the SAC condition were better than that in the aided FAC condition. There was no significant difference in temporal modulation thresholds between unaided and aided SAC conditions. The * symbol means statistically significant.

The mean unaided and aided modulation thresholds were reported in Figure 3. An independent *t*-test showed that the unaided modulation threshold and the aided modulation thresholds measured in the SAC were better than that using FAC. There was no significant difference in modulation thresholds between the unaided and aided SAC conditions. Thus, the acoustic changes resulting from different compression speeds can influence an individual's measured temporal resolution.

### Speech in Noise Performance

**Figure 4:**
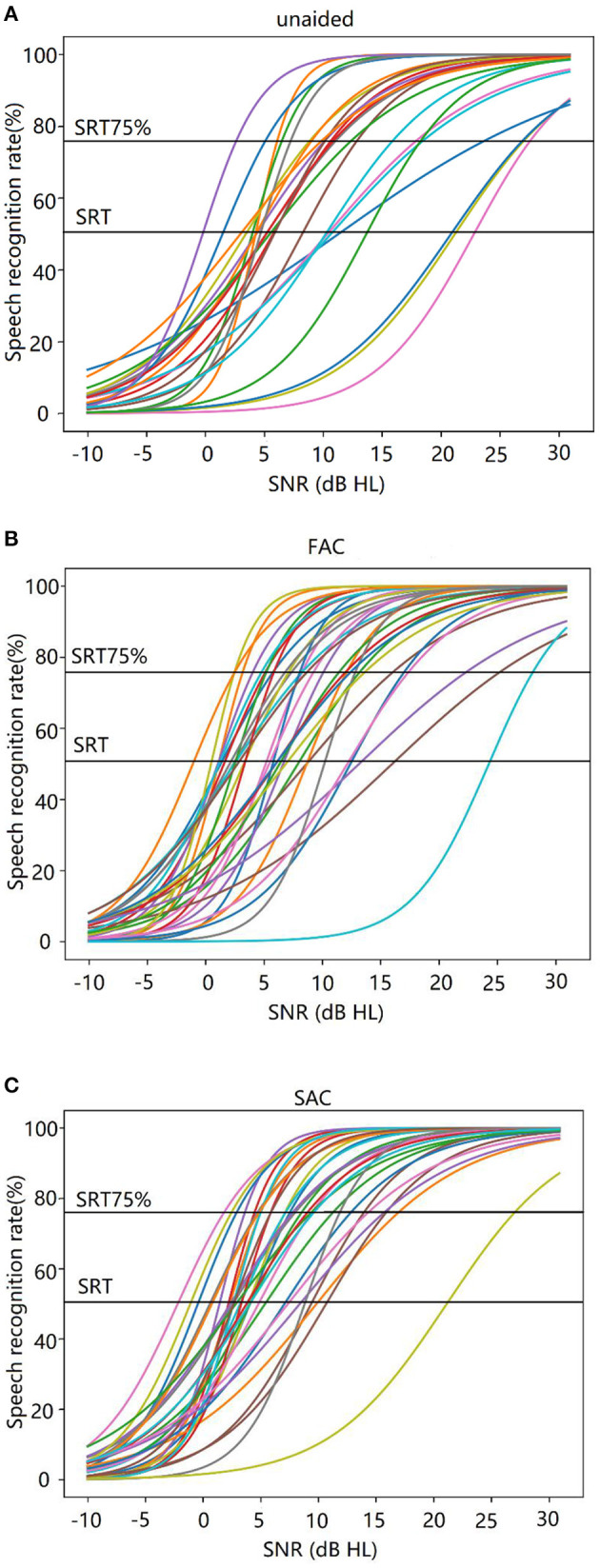
**(A–C)** Individual logistic functions (or performance-intensity functions) estimated across signal-to-noise ratios. **(A)** Unaided, **(B)** FAC, and **(C)** SAC.

Logistic functions were fitted to each individual subject's data at the 4 individualized SNRs for each test condition. In generating the logistic functions, we assigned a performance of 0% when the SNR was −10 dB and 100% when the SNR was 30 dB. The generated logistic functions, which represent the subjects' performance across a range of SNRs for each hearing aid condition (unaided, FAC, SAC), are summarized in Figures 4A–C.

Using this function, we estimated the speech reception threshold (SRT) at a criterion level of 75% (SRT75%) to reflect more closely on the SNR condition that subjects needed for successful daily communication in real life (Smeds et al., 2015; Wu et al., 2018; Kuk et al., 2019).

**Figure 5:**
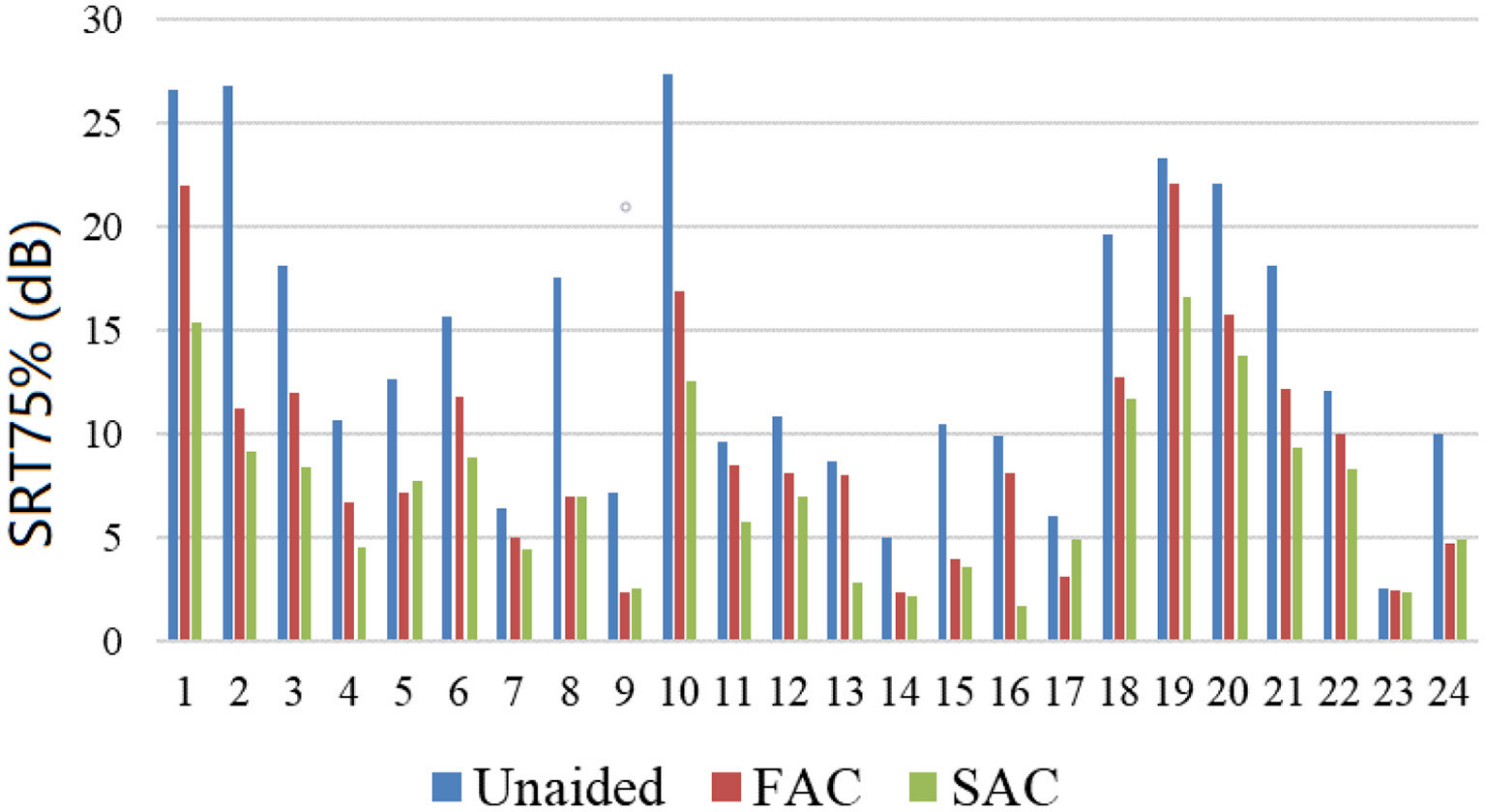
SRT75% measured across participants.

**Figure 6:**
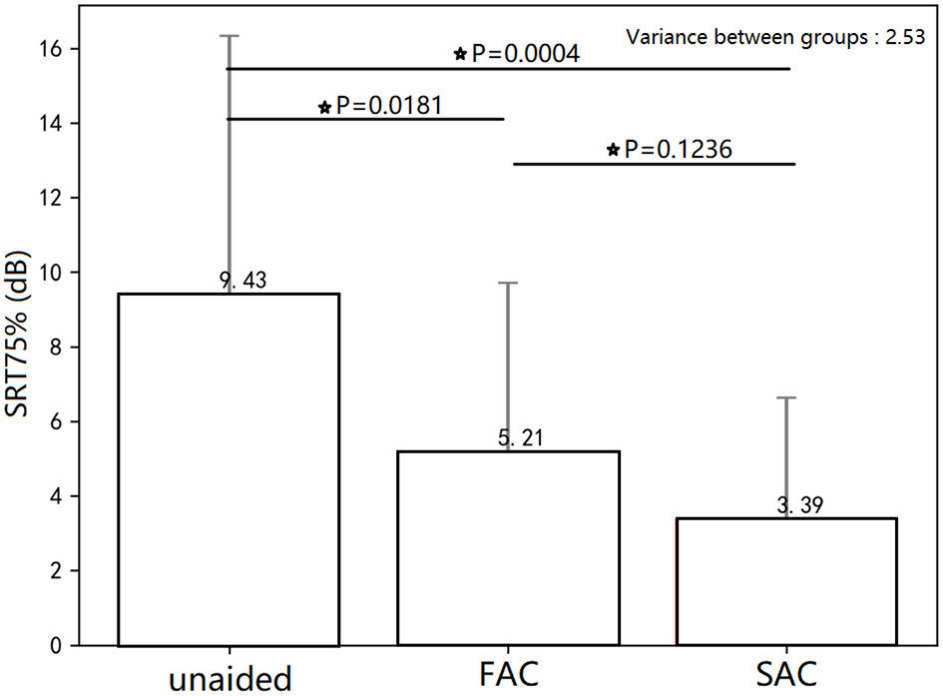
Mean SRT75% for the three conditions. Speech recognition in noise wearing either hearing aid was significantly improved over the unaided condition. There was no significant difference in SRT75% between the two hearing aids. This * symbol means statistically significant.

The individual SRT75% is summarized in Figure 5. The average SRT75% for each hearing aid condition (unaided, SAC, FAC) is shown in Figure 6. The mean unaided SRT75% was 9.43 dB. The mean SRT75% with FAC was 5.21 dB, while the mean SRT75% with SAC was 3.39 dB. A smaller SRT75% reflects better speech in noise performance. An independent *t*-test showed that the speech recognition in noise wearing either hearing aid was significantly improved over the unaided condition. However, there was no significant difference in performance between the two hearing aids. Thus, compression speeds did not significantly affect speech recognition in noise.

### Decision Tree Analysis of Compression Speed Candidacy

Both FAC and SAC hearing aids significantly improved the listeners' speech recognition of noise, but there was not a significant SRT75% difference between the two forms of compression speeds. While on a group level that may be the case, the varied unaided temporal resolution and speech in noise abilities of the presbycusic listeners suggest the possibility that compression speed may need to be customized to the individual's residual auditory abilities for maximum or optimal benefits. Thus, we turned to the use of a decision tree analysis (using python3.8 scikit-learn DecisionTreeClassifier). A decision tree is a supervised learning algorithm intended to produce a classification for a new object whose characteristics are known. Every inner node of the tree is labeled as a test, which compares the input attribute to a threshold, and every terminal node is labeled as a category. To obtain a classification for a new object whose attribute values are known, we could put it in the tree from the top node. When a terminal node is reached, the object's classification would have been defined (Geurts et al., 2009). In this analysis, subject performance in the unaided mode (along with the AP) was used as criterion measures to direct the selection of the optimal compression speeds. A detailed description of each step in the decision tree is provided.

First, we determined which compression speed may be more optimal for each subject. We measured the ratio of the aided SRT75% using FAC (fSRT75%) and that using SAC (sSRT75%) to compare their relative efficacy. As the 95% confidence interval on the SRT75% data was estimated at 0.156, 1 ± 0.156 was taken as the upper and lower limit of the confidence interval. Thus, when fSRT75%/sSRT75% > 1+0.156, SAC yielded a lower SRT and was judged better. When fSRT75%/sSRT75% < 1–0.156, FAC yielded a lower SRT and was thus better. When 1 + 0.156 ≥ fSRT75%/sSRT75% ≥ 1–0.156, the efficacy of the two hearing aids was judged similar. Based on this analysis, 1 subject performed better (ratio < 0.844) with FAC, 12 subjects performed better with SAC (ratio > 1.156), and 11 subjects had similar hearing performance between two compression speeds (ratio between 0.844 and 1.156). This finding may also explain the non-significant difference in SRT75% measured between SAC and FAC.

**Figure 7:**
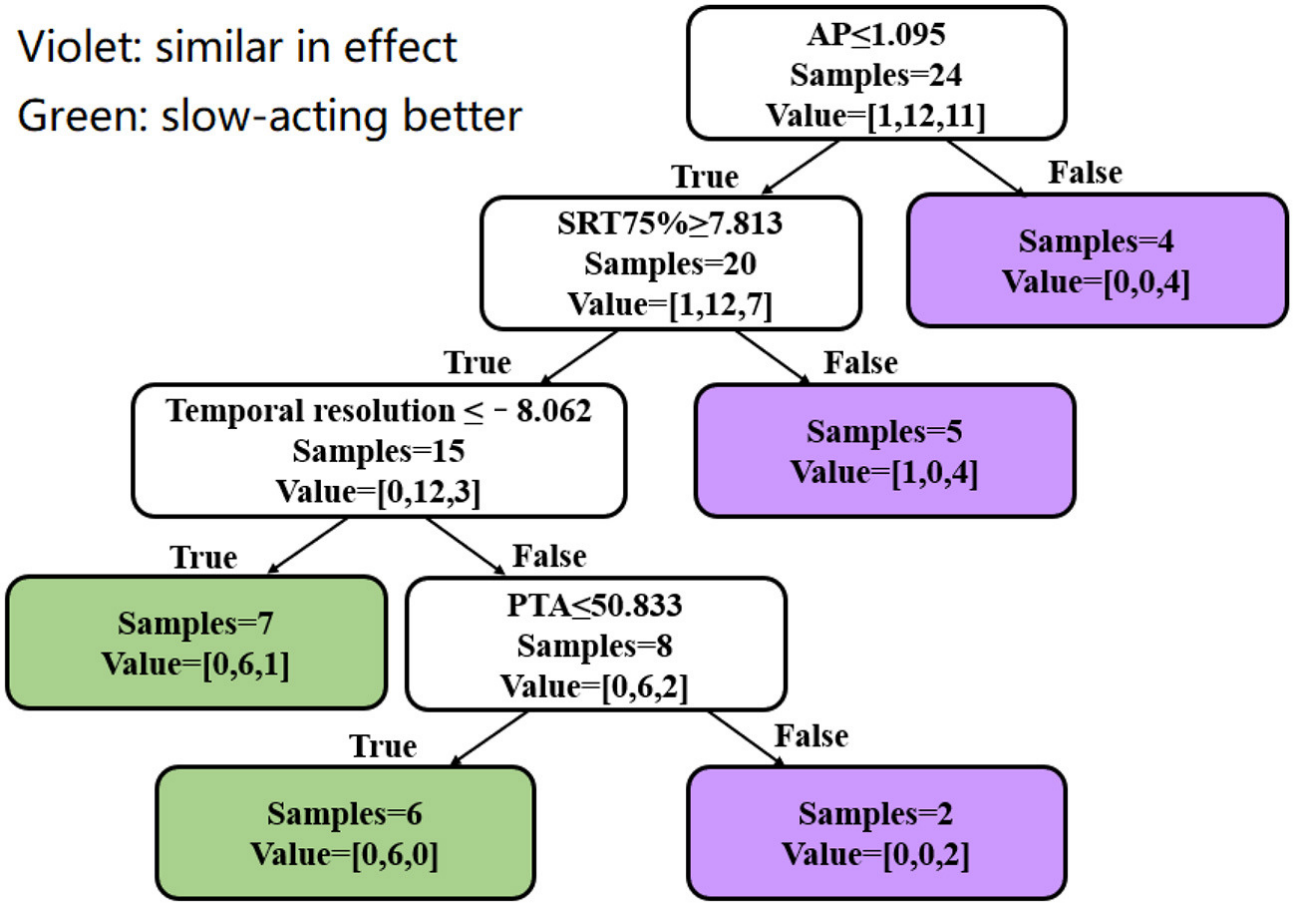
Decision tree analysis. Violet represents a similar aided SRT75% between the two compression speeds. Green represents better SRT75% for SAC than FAC. Value = [A, B, C]: A is the number of subjects who performed better with FAC, B is the number of subjects who performed better with SAC, and C is the number of subjects where performance between FAC and SAC was similar.

The decision tree analysis takes known factors that may affect the decision as inputs to generate the steps. According to previous studies, factors that may affect hearing aid performance include gender, age, cognitive level, cochlear ECochG (-SP/AP), SP, AP, PTA, unaided temporal resolution, and unaided SRT75%. These data were input to the model and the most significant factors that affected the decision included PTA, AP value, unaided modulation thresholds, and unaided SRT75%. The probable reasons for these factors were explained earlier in the methods. Of the 24 subjects included in the decision tree, 22 were correctly identified. The decision tree is shown in Figure 7.

The decision tree starts with the individual's AP. If the individual's AP is >1.095 μV, the aided SRT75% obtained with SAC and FAC would be similar. Four subjects were identified in this step. When AP ≤ 1.095 μV, the unaided SRT75% should be further compared. When the unaided SRT75% is <7.813 dB, the aided SRT between SAC and FAC should be similar. Five subjects were identified in this step with 4 showing no difference in aided SRT75% between SAC and FAC and 1 better aided SRT75% using FAC. If the unaided SRT75% is ≥7.813 dB, one should proceed to examine the unaided modulation thresholds. When the unaided modulation threshold is ≤-8.062 dB, SAC should yield a better SRT75% than FAC. Seven subjects were identified, of which 6 had a better aided SRT75% with SAC, while the remaining subject performed equally well with SAC and FAC. On the contrary, if the unaided modulation threshold is >-8.062 dB, the impact of hearing loss emerged. When the PTA is ≤ 50.833 dB, SAC yielded a better aided SRT75% than FAC. Six subjects were included in this step, and all had a lower SRT75% with SAC (than FAC). If the PTA is >50.833 dB, the effect of compression speed should be similar. Two subjects were included in this step, and both showed similar aided SRT75% for SAC and FAC.

### Preliminary Validation Study on the Decision Tree Analysis

To verify the validity of the decision tree, 10 additional patients were enrolled (5 male patients and 5 female patients with an age range between 55 and 86 years). Their MoCA scores ranged from 21 to 28, and PTA ranged between 41.7 and 60 dB HL. The same experimental procedure, as mentioned earlier, was followed. The demographic characteristics and audiometric results of subjects are shown in Table 2.

**Table 2:** Demographic characteristics and audiometry results of 10 additional subjects.

Age (year), median (Q1, Q3)	76.8, 78.5 (70.5, 83.8)
Male, *n* (%)	5 (50)
PTA (dB HL), median (Q1, Q3)	53.8, 55 (52.5, 56.3)
MoCA (point), median (Q1, Q3)	25.6, 25.5 (25, 27)
ECochG (-SP/AP %), median (Q1, Q3)	46.5, 43 (37.6, 56)
AP, median (Q1, Q3)	0.693, 0.685 (0.485, 0.84)

**Figure 8:**
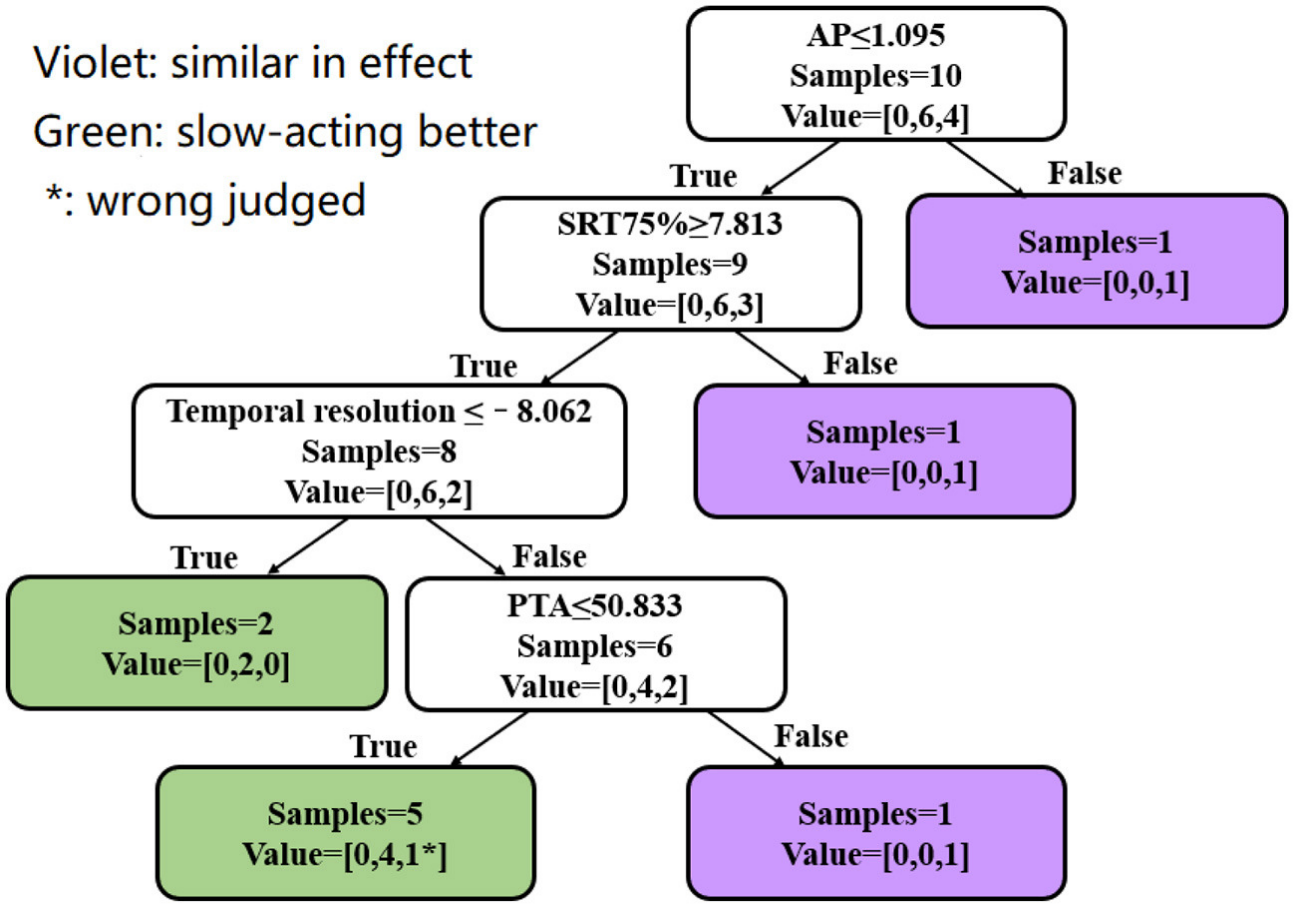
Verification of the decision tree model. Violet represents a similar aided SRT75% between the two compression speeds. Green represents better SRT75% for SAC than FAC. “*” Represents a subject who was judged incorrectly. Value = [A, B, C]: A is the number of subjects who performed better with FAC, B is the number of subjects who performed better with SAC, and C is the number of subjects where performance between FAC and SAC was similar.

The data of these 10 subjects were included in the decision tree for verification. Nine subjects were correctly identified by the decision tree, with a pass rate of 90%. The judgment process and results are shown in Figure 8.

## Discussion and Conclusion

This study showed that the unaided temporal modulation thresholds were preserved using SAC but were degraded using FAC. Aided speech recognition threshold using a 75% criterion (SRT75%) was significantly better than the unaided SRT75%, but not so between SAC and FAC. The unaided SRT75%, unaided modulation thresholds, along with AP and PTA allowed us to perform a decision tree analysis that may help select the optimal compression speed for an individual listener reliably, using the aided SRT75% as a criterion of performance.

The unaided modulation thresholds reflect the auditory system's ability to code temporal information. Aging and/or hearing loss alone or in combination can further damage this ability and make decoding of such temporal information difficult. In this study, we have shown that the compression speed could further alter or degrade such ability. In particular, the use of FAC smears the temporal contrast between louder and softer parts of the input sounds to result in poorer modulation thresholds. The use of SAC, on the contrary, preserved the modulation threshold measured in the unaided mode. As modulation thresholds have been reported to reflect speech in noise ability, it would suggest that speech in noise ability, measured as the SNR to reach 75% correct identification, would be poorer with FAC than with SAC. While better SRT75% was observed with SAC than FAC, the difference was non-significant.

A plausible reason for the non-significant difference between SAC and FAC may be the mixture of subjects with different residual temporal resolution abilities in this study. If their residual ability is either good or poor, the results of the comparison may be more clear-cut in showing the advantage of one form of compression speed over the other. Indeed, the decision tree analysis revealed that only 11 subjects showed a clearer benefit with SAC and 1 subject showed a clearer benefit with FAC. Notably, twelve subjects were indifferent in their performance between SAC and FAC. Fewer subjects showing indifferent results or more subjects showing performance benefits with one compression speed would likely change our observations and conclusions.

In that regard, the decision tree analysis provided good insights into the choice of optimal compression speeds on an individual basis. Rather than concluding that one form of compression speed is universally optimal for all listeners with presbycusis, the decision tree allows one to consider various individual factors (such as AP, PTA, unaided SRT75%, and unaided modulation threshold) in order to select the most optimal compression speed on an individual basis. If the values reported in this decision tree are further validated in future studies, this could offer an avenue for finer selection of hearing aid parameters and ultimately further improve elderly patients' speech in noise performance with their hearing aids.

In this decision tree, AP is taken as the first judgment criterion. Patients with high AP amplitude have a similar SRT75% between FAC and SAC, while in some patients with lower AP, SAC can achieve a better SRT75%. The low AP amplitude of ECochG reflects cochlear synaptopathy or low SR fiber dysfunction and is related to speech perception dysfunction in presbycusic patients. A study found that SAC can attain better speech recognition for children with auditory neuropathy, whose main lesion site is the auditory synapse (Narne and Vanaja, 2008). So, we inferred that SAC may facilitate signal transmission from inner hair cells to a nerve fiber in cochlear synaptopathy or low SR fiber dysfunction. Therefore, we reasonably suggest that among the patients with AP ≤ 1.095 μV, some of them have cochlear synaptopathy or auditory nerve dysfunction which may have been better served using SAC.

While we are motivated by the current findings of this study, we also recognized certain limitations to this study. First is the small sample size of patients (*n* = 24) that we used. Additional patients could increase the power of our observations and likely provide more robust criterion cutoff values in our decision tree analysis. Second, compression speed is only one important parameter in a compression hearing aid. The compression ratio, or the availability of other signal processing algorithms like noise reduction and directional microphones, could also affect the reported benefit and/or satisfaction toward the hearing aids. A more robust decision tree may also consider those factors. Third, our criterion of “more benefit” is simply the ratio of the SRT75% measured with FAC and SAC. Indeed, a lower SRT75% reflects better speech understanding of noise and is a good measure of benefit. However, other measures of performance such as sound quality, or an SRT at other criteria such as 50% or 90%, may also be a good metric to examine relative performance/benefit. Finally, all participants in this study had moderate-to-severe hearing loss. This may limit the generalizability of the results of the decision tree to other degrees of hearing loss. More subjects with varied hearing levels may be helpful to determine the impact of hearing loss (PTA) on the decision.

## Data Availability Statement

The data used to support the findings of this study were supplied by Peking University First Hospital under license and so cannot be made freely available. Requests for access to these data should be made to M.D. Zhang (yizhangsyx@163.com).

## Ethics Statement

The studies involving human participants were reviewed and approved by Biomedical Research Committee of the Peking University First Hospital. The patients/participants provided their written informed consent to participate in this study.

## Author Contributions

YL, YiZ, and JC conceived the study. YL, YiZ, JC, and YaZ developed the study design. YiZ, YaZ, and BS conducted the data wrangling. YL and YiZ drafted the article and conducted the analysis. YL, YiZ, and BS made important contributions to the interpretation of data. All authors contributed to the article and approved the submitted version.

## Funding

This project was supported by the Beijing Municipal Science & Technology Commission (No. Z191100006619027) and the National Natural Science Foundation of China (Grant No: 82071070).

## Conflict of Interest

BS is employed by Widex Hearing Aid Shanghai Co., Ltd. The remaining authors declare that the research was conducted in the absence of any commercial or financial relationships that could be construed as a potential conflict of interest.

## Publisher's Note

All claims expressed in this article are solely those of the authors and do not necessarily represent those of their affiliated organizations, or those of the publisher, the editors and the reviewers. Any product that may be evaluated in this article, or claim that may be made by its manufacturer, is not guaranteed or endorsed by the publisher.
